# Targeting the cross-talk between Urokinase receptor and Formyl peptide receptor type 1 to prevent invasion and trans-endothelial migration of melanoma cells

**DOI:** 10.1186/s13046-017-0650-x

**Published:** 2017-12-08

**Authors:** Concetta Ragone, Michele Minopoli, Vincenzo Ingangi, Giovanni Botti, Federica Fratangelo, Antonello Pessi, Maria Patrizia Stoppelli, Paolo Antonio Ascierto, Gennaro Ciliberto, Maria Letizia Motti, Maria Vincenza Carriero

**Affiliations:** 1 IRCCS Istituto Nazionale Tumori ‘Fondazione G. Pascale’, Naples, Italy; 2 University of Campania “Luigi Vanvitelli”, Naples, Italy; 3 Peptipharma, Viale Città D’Europa 679, 00144 Rome, Italy; 4 Institute of Genetics and Biophysics, National Research Council, Naples, Italy; 5 IRCCS Istituto Nazionale Tumori Regina Elena, Rome, Italy; 6 University ‘Parthenope’, Via Acton 38, 80133 Naples, Italy

**Keywords:** Urokinase receptor, Formyl peptide receptor type 1, Melanoma, Peptides, Trans-endothelial migration

## Abstract

**Background:**

Accumulating evidence demonstrates that the Urokinase Receptor (uPAR) regulates tumor cell migration through its assembly in composite regulatory units with transmembrane receptors, and uPAR_88–92_ is the minimal sequence required to induce cell motility through the Formyl Peptide Receptor type 1 (FPR1). Both uPAR and FPR1 are involved in melanoma tumor progression, suggesting that they may be targeted for therapeutic purposes. In this study, the role of the uPAR-FPR1 cross-talk to sustain melanoma cell ability to invade extracellular matrix and cross endothelial barriers is investigated. Also, the possibility that inhibition of the uPAR mediated FPR1-dependent signaling may prevent matrix invasion and transendothelial migration of melanoma cells was investigated.

**Methods:**

Expression levels of uPAR and FPR1 were assessed by immunocytochemistry, Western Blot and qRT-PCR. Cell migration was investigated by Boyden chamber and wound-healing assays. Migration and invasion kinetics, trans-endothelial migration and proliferation of melanoma cells were monitored in real time using the xCELLigence technology. The agonist-triggered FPR1 internalization was visualized by confocal microscope. Cell adhesion to endothelium was determined by fluorometer measurement of cell-associated fluorescence or identified on multiple z-series by laser confocal microscopy. The 3D–organotypic models were set up by seeding melanoma cells onto collagen I matrices embedded dermal fibroblasts. Data were analyzed by one-way ANOVA and post-hoc Dunnett t-test for multiple comparisons.

**Results:**

We found that the co-expression of uPAR and FPR1 confers to A375 and M14 melanoma cells a clear-cut capability to move towards chemotactic gradients, to cross extracellular matrix and endothelial monolayers. FPR1 activity is required, as cell migration and invasion were abrogated by receptor desensitization. Finally, melanoma cell ability to move toward chemotactic gradients, invade matrigel or fibroblast-embedded collagen matrices and cross endothelial monolayers are prevented by anti-uPAR_84–95_ antibodies or by the RI-3 peptide which we have previously shown to inhibit the uPAR_84–95_/FPR1 interaction.

**Conclusions:**

Collectively, our findings identify uPAR and FPR1 as relevant effectors of melanoma cell invasiveness and suggest that inhibitors of the uPAR_84–95_/FPR1 cross-talk may be useful for the treatment of metastatic melanoma.

**Electronic supplementary material:**

The online version of this article (10.1186/s13046-017-0650-x) contains supplementary material, which is available to authorized users.

## Background

Melanoma, due to its tendency to metastasize through the lymphatic and blood vessels, is the most aggressive skin cancer, and its incidence has dramatically risen during the last half century [1]. Although most melanoma cases are early diagnosed and surgically resected, the later stages had still very poor survival rates because of the lack of effective therapies [2]. In recent years, targeted treatments are allowing to overcome the ineffectiveness of the conventional therapies and achieve an impressive improvement of the patients survival [3, 4]. However, resistance and clonal expansion produced by the main targeted inhibitors develop in few months as a consequence of the activation of alternative proliferation-inducing pathways [5, 6]. The scenario is changing in recent years due to the advent of immunotherapy [7]. However, it has to be noted that immunotherapy is effective only in a subset of patients [8]. Therefore, the scientific community is driven to identify new targets molecules in order to develop new therapeutic strategies.

Several studies support the important role of the plasminogen activator system in this tumor type. Expression of urokinase (uPA) correlates with the metastatic potential of melanoma cells and the expression of uPA and its cognate receptor (uPAR) are increased in late stage melanocytic tumors [9, 10]. Other studies support a direct involvement of uPAR in the melanoma progression. Hypoxia promotes lymph node metastasis in human melanoma xenografts by up-regulating uPAR [11] and inhibition of uPAR by RNA interference has been reported to reduce tumor growth in human melanoma skin and exert pro-apoptotic effects in melanoma cells with acquired resistance to B-RAFi and MEKi [12, 13].

The uPAR consists of three domains (D1, D2, and D3), anchored to the cell surface through a carboxy terminal glycosyl-phosphatidyl-inositol anchor [14]. When expressed on cell surface, uPAR promotes cell associated proteolysis by binding to uPA, which locally converts plasminogen into active plasmin, thus favoring tissue invasion and metastasis [15, 16]. Ligand-engaged uPAR also acts as a potent regulator of cell migration and matrix attachment, independently of the uPA catalytic activity [15, 16]. We and others have shown that uPAR signaling occurs through its assembly in composite regulatory units with extracellular matrix (ECM) proteins such as vitronectin, and transmembrane receptors, including the G protein-coupled formyl-peptide receptors (FPRs) [17–24]. Due to the pleiotropic nature of its interactors, uPAR represents both a challenge and an opportunity for drug discovery. However, despite significant effort, no uPAR-targeted therapeutics are in clinical evaluation to date. This encourages innovative, therapeutic approaches devoted to interfering with uPAR/co-receptor interactions. The uPAR domains D1-D3 are connected by short linker regions [25]. D1-D3 pack together into a concave structure that shifts to an active conformation upon binding to uPA [26, 27]. The linker between D1-D2 is more flexible than that between the D2-D3 domains [27–29], and includes the protease-sensitive crucial signaling region, uPAR_84–95_ [30]. In the form of a synthetic peptide, the minimal 88–92 sequence (Ser^88^-Arg-Ser-Arg-Tyr^92^, SRSRY) retains chemotactic activity and triggers directional cell migration and angiogenesis in vitro and in vivo [20–22, 30, 31]. Mechanistically, these activities are mediated by the interaction of uPAR with the formyl-peptide receptor type 1 (FPR1) which, in turn, activates the vitronectin receptor with an inside-out type of mechanism which involves PKC and ERK phosphorylation [22]. FPRs are a family of 7 transmembrane domain, Gi-protein-coupled receptors that exert multiple functions in many pathophysiologic processes because of their capacity to interact with a variety of structurally diverse ligands [32]. Human FPR1, originally identified in neutrophils, monocytes and macrophages, elicits many responses upon ligation of formyl-peptide ligands derived from bacteria and/or mitochondria of eukaryotic cells, including morphological polarization, locomotion, production of reactive-oxygen species and release of cytokines and proteolytic enzymes [33]. In recent years, FPR1 has been shown to be expressed also in several non-myelocytic cells, and accumulating evidence demonstrates that FPR1 is involved in progression of solid tumors [34–37]. FPR1 is overexpressed in human primary melanoma and associates with aggressive phenotype [35].

Therefore, the inhibition of the uPAR mediated FPR1-dependent signal represents an attractive target to inhibit the metastatic process in solid tumors. We previously showed that the substitution of Ser90 with a glutamic acid residue in the uPAR_84–95_ chemotactic sequence prevents agonist-triggered FPR1 activation and internalization [38]. Following this observation, we developed a series of linear peptides containing substitution of Ser90 with a glutamic acid or an α-aminoisobutyric acid (Aib) residue in the Ser^88^-Arg-Ser-Arg-Tyr^92^ sequence that inhibit the uPAR/FPR1 interaction and reduce to basal levels directional cell migration, invasion and angiogenesis [39–42]. To generate more stable uPAR/FPR1 inhibitors, we applied the Retro-Inverso (RI) approach [43] to our previously described uPAR/FPR1 inhibitors [39–42]. The retro-inverso peptide RI-3, was selected as the best inhibitor the uPAR mediated FPR1-dependent signal. RI-3 is stable in human serum and has no effect on cell proliferation, even at a 10 μM concentration. At nanomolar concentrations, it inhibits migration, matrigel invasion and trans-endothelial migration of human sarcoma cells. Moreover, when administered in mice bearing sarcomas, RI-3 reduced tumor growth, intra-tumor microvessel density and vascular infiltration by tumor cells [44].

In this study we explored the effects of uPAR-FPR1 complexes on melanoma progression. We found that the co-expression of uPAR and FPR1 confers to melanoma cells the capability to move towards chemotactic gradients, to cross ECM and endothelial monolayers. The important role of the uPAR_84–95_ sequence in determining the invasive ability of melanoma cells has been confirmed by the finding that anti-uPAR_84–95_ antibodies as well as RI-3 peptide counteract the migratory ability and invasiveness of melanoma cells. These findings identify uPAR/FPR1 complexes as novel therapeutic targets in melanoma and suggest that inhibitors of the uPAR_84–95_/FPR1 interaction may be useful for the treatment of metastatic melanoma.

## Methods

### Cell lines

Human melanoma A375 and M14 cell lines were purchased from ATCC*.* The human melanoma cell line A375M6, isolated from lung metastasis of SCID^bg/bg^ mice i.v. injected with human melanoma A375P cells [45], was kindly provided by Prof. Gabriella Fibbi (Department of Experimental and Clinical Biomedical Science, University of Florence, Florence, Italy). A375 cells were cultured in RPMI whereas A375M6 and M14 cells were cultured in DMEM. In all cases, media were supplemented with 10% fetal bovine serum (FBS), penicillin (100 μg/mL), streptomycin (100 U/ml) and maintained at 37 °C in a humidified atmosphere of 5% CO_2_. Human Umbilical Vein Endothelial Cells (HUVEC)s, purchased by Lonza, were employed between the third and the seventh passage and grown in Eagle Basal Medium supplemented with 4% FBS, 0.1% gentamicin, 1 μg/mL hydrocortisone, 10 μg/mL epidermal growth factor and 12 μg/mL bovine brain extract (Cambrex). Normal human dermal fibroblasts (NHDF) purchased by Lonza were cultured in Fibroblast Basal Medium supplemented with 2% FBS, penicillin (100 μg/mL), streptomycin (100 U/ml), 1 ml/L insulin, 1 ml/L human fibroblast growth factor-B, 1:1000 ratio gentamicin, 15 μg/ml amphotericin and maintained at 37 °C in a humidified atmosphere of 5% CO_2_.

To prepare conditioned media, A375 and A375 M6 cells (1.5 × 10^6^ cells/well) were seeded on 6-well plates in growth medium. After 6 h, medium was removed and cells, after extensive washing with PBS, were incubated with 1.5 mL serum-free medium. After 18 h, the medium was recovered, cleared by centrifugation and concentrated 30 times by Amicon Ultra centrifugal filters 10 K (Millipore).

### Plasmids and transfections

A375 transfectants, stably expressing Green Fluorescent Protein (GFP), were obtained using pEGFP-N1 vector (Clontech) and polyfectamin transfection reagent (Quiagen). Geneticin-resistant cells expressing the highest levels of GFP under fluorescence microscopy were isolated and amplified. The expression vector pcDNA3-uPAR was constructed by inserting the 1027 bp EcoRI-EcoRI fragment from pBluescript II SK, containing the whole human uPAR-cDNA as previously described [46]. The sequence was confirmed by DNA sequencing. The empty pcDNA3 and pcDNA3-uPAR vectors were transfected into M14 cells using HiPerFect transfection reagent, according to the manufacturer’s specifications (Qiagen). Five clones were isolated by limiting dilution in the presence of G418 selection (1.5 mg/mL Geneticin) and then cultured in the presence of 0.8 mg/mL Geneticin.

siRNA targeting uPAR were purchased by Qiagen (SI03033289). A randomized sequence (All star negative controlsiRNA, SI03650318) was used as negative RNA control. A375 cells (6 × 10^5^ cells/sample) were exposed to the transfection mixture containing 5 nM siRNA diluted in RPMI and HiPerfect (Qiagen) for 96 h. Transfection mixture was refreshed after 48 h.

### Fluorescence microscopy

Cells (~2 × 10^4^/sample) were seeded on glass coverslips and cultured for 24 h in growth medium. Then, slides were washed with PBS, fixed with 2.5% formaldehyde in PBS for 10 min at 4 °C and incubated for 1 h at 4 °C with 2 μg/mL R4 anti-uPAR monoclonal antibody or rabbit anti-1:100 anti-FPR1 antibody (#113531Ab, Abcam). Then, 1:700 goat Alexa Fluor 488 anti-rabbit IgG or rabbit Alexa Fluor 488-conjugated F(ab’)2 fragment of anti-mouse IgG (Molecular Probes) were applied to slides at 23 °C for 40 min. Nuclear staining was performed with 4–6-diamidino-2-phenylindole dye (DAPI). To visualize the cytoskeleton, cells were fixed with 2.5% formaldehyde, permeabilized with 0.1% Triton X-100 for 10 min at 4 °C, and incubated with 0.1 μg/mL rhodamine-conjugated phalloidin (Sigma-Aldrich) for 40 min. To analyze agonist-dependent FPR1 internalization, cells grown on glass slides were exposed to 10 nM N-formyl-Nle-or Leu-Phe-Nle-Tyr-Lys-fluorescein (Molecular Probes), diluted in serum-free DMEM for 30 min at 37 °C as described [39, 40]. In all cases, coverslips were mounted using 20% (*w*/*v*) Mowiol, visualized with the the Axiovert 200 M fluorescence inverted microscope connected to a video-camera or with the 510 META-LSM confocal microscope (Carl Zeiss).

### Quantitative real-time PCR analysis

Total cellular RNA was isolated by lysing cells with TRIzol solution according to the manufacturer’s instructions. RNA was precipitated and quantitated by spectroscopy. Five micrograms of total RNA were reversely transcribed with random hexamer primers and 200 U of EuroScript-Euroclone reverse transcriptase. uPAR expression in all melanoma cell lines was determined by a quantitative Real-Time PCR with an Applied Biosystem 7900 Fast Real Time PCR System (Applied Biosystems) and determined by the comparative Ct method using GAPDH as the normalization gene. Amplification was performed with the default PCR setting: 40 cycles of 95 °C for 15 s and of 60 °C for 60 s using SYBR Green–select master mix (Applied Biosystem). Primers used for RT-PCR were as follows: uPAR: sense, 5′- GCCCAATCCTGGAGCTTGA-3; antisense, 5′-TCCCCTTGCAGCTGTAACACT-3′; GAPDH: sense,5′-GAC AGT CAG CCG CAT CTT CT-3′ antisense, 5′-TTA AAA GCA GCC CTG GTG AC-3′.

### Western blot

Cells detached using 200 mg/L EDTA, 500 mg/L trypsin (Cambrex), were lysed in RIPA buffer (10 mM Tris pH 7.5, 140 mM NaCl, 0.1%SDS, 1% Triton X-100, 0.5% NP40) containing protease inhibitor mixture. Protein content of cell lysates was measured by a colorimetric assay (BioRad). 40 μg proteins or 50 μl concentrated conditioned medium from A375 or A375M6 cells were separated on 10% SDS-PAGE and transferred onto a polyvinylidene fluoride membrane. In all cases, the membranes were blocked with 5% non-fat dry milk and probed with 1 μg/mL R4 anti-uPAR monoclonal antibody recognizing uPAR D3 domain, 1 μg/mL anti-FPR1 polyclonal antibody (#128296 Ab, Abcam), 0.2 μg/mL GAPDH Ab (Santa Cruz Biotechnology), or 1 μg/mL 389 anti-uPA polyclonal antibody (American Diagnostica). Washed filters were incubated with horseradish peroxidase-conjugated anti-mouse or anti-rabbit antibody and detected by ECL (Amersham- GE Healthcare). Densitometry was performed using the NIH Image 1.62 software (Bethesda,MD). Each experiment was performed three times.

### Peptide synthesis

The peptide RI-3 was custom-synthesized on solid-phase with Fmoc/t-Bu chemistry (IRBM Science Park, Pomezia (Rome) Italy). RI-3 was purified by reversed-phase HPLC using water/acetonitrile gradients, and characterized by UPLC-MS [44].

### Cell proliferation

Cell proliferation was assessed using E-16-well plates and the xCELLigence Real Time Cell Analysis (RTCA) technology (Acea Bioscience) as described [47]. Briefly, cells (2 × 10^3^/well) were seeded in 16-well plates in growth medium and left to growth for 72 or 96 h. Microelectrodes placed on the bottom of plates, detect impedance changes which are proportional to the number of adherent cells and are expressed as Cell Index. The impedance value of each well was automatically monitored by the xCELLigence system and expressed as a Cell Index value. Doubling times for each cell clone were calculated from the cell growth curve during the exponential growth. The experiments were performed twice in quadruplicate.

### Wound-healing assay

For wound-healing assays, confluent cells grown in a 24 multi-well plate were wounded with a sterile pipette tip and exposed to growth medium. One field/dish including the scratched path was selected and scanned sequentially every 30 min for 24 h. The extent of wounded areas was evaluated by the Axiovision 4.8 software and plotted against time. Data points were fitted with a linear equation whose slope represents the cell speed. All experiments were performed in triplicates.

### Cell migration and invasion in Boyden chambers

Chemotaxis assays were performed in Boyden chambers, using 8 μm pore size PVPF-filters (Nucleopore) as previously described [22]. Briefly, 1 × 10^5^ viable cells were seeded in each upper chamber in serum-free medium. The lower chamber was filled with serum-free medium containing diluents, 10% FBS or 10 nM SRSRY peptide as chemoattractants. In some experiments, 10 nM RI-3, 2 μg/ml 399 anti-uPAR (American Diagnostica), 2 μg/ml anti-uPAR_84–95_ purchased by PRIMM and recognizing the uPAR_84–95_ sequence [31] or 2 μg/ml anti-α-tubulin (Cell Signalling) polyclonal antibodies, were pre-incubated with the cell suspension for 1 h at 37 °C and kept throughout the assay. Other experiments were performed using cells desensitized with 100 nM fMLF or 100 nM SRSRY for 1 h at 37 °C in humidified air with 5%CO_2_ as described [22, 31]. Cells were allowed to migrate for 4 h at 37 °C, 5% CO_2_. For the invasion assays, filters were coated with 50 μg/filter matrigel (BD Biosciences) and cells (3x10^4^viable cells/well) were allowed to invade matrigel for 18 h at 37 °C, 5% CO_2_. In all cases, at the end of the assay, cells on the lower filter surface were fixed with ethanol, stained with haematoxylin and 10 random fields/filter were counted at 200× magnification. The arbitrary value of 100% was given to the basal cell migration or invasion assessed in the absence of chemoattractant. All experiments were performed three times in triplicate, and the results were expressed as percentage of the basal cell migration or invasion.

### Migration kinetic of cells monitored in real time

Kinetic of cell migration was monitored in real time using the xCELLigence RTCA technology as described [44, 47]. For these experiments we used CIM-16-well plates which are provided with interdigitated gold microelectrodes on bottom side of a filter membrane interposed between a lower and an upper compartment. The lower chamber was filled with serum-free medium or chemoattractans diluted serum-free medium with/without 10 nM RI-3 or 2 μg/ml the indicated antibodies. Cells (2 × 10^4^ cells/well) were seeded on filters in serum-free medium. Microelectrodes detect impedance changes which are proportional to the number of migrating cells and are expressed as cell index. Migration was monitored in real-time for at least 12 h. Each experiment was performed at least twice in quadruplicate.

### Invasion kinetic of cells monitored in real time

This assay was performed using E-16-well plates and the xCELLigence RTCA technology as described [44, 47]. Bottom wells were coated with 20 μg/well matrigel diluted in serum-free medium. Matrigel was allowed to polymerize for 1 h at 37 °C prior to seeding cells (1 × 10^4^ cells/well) suspended in in serum-free medium (CTRL) or growth medium plus/minus 10 nM RI-3 or 2 μg/ml the indicated antibodies. Cells that cross matrigel adhere to the bottom of plates causing impedance changes which are proportional to the number of invading cells. Matrigel invasion was monitored in real-time for 20 h. The impedance value of each well was automatically monitored and expressed as a cell index value. Slopes represent the change rate of cell index generated in a 1–18 h time frame. The experiments were performed three times in in quadruplicate.

### Cell adhesion onto endothelium

GFP-tagged A375 cells were seeded on an endothelial monolayer as previously described [44]. Briefly, sterile round glass coverslips (12 mm in diameter) were coated with 1:8 diluted matrigel. HUVEC (5 × 10^4^ cells in 200 μL/well) were plated onto matrigel and allowed to form a monolayer for ~24 h at 37 °C, 5% CO_2_ prior to seeding GFP-A375 cells (1.5 × 10^4^ cells/well) suspended in complete endothelial medium plus diluents or 10 nM RI-3. At the indicated times, plates were accurately washed with PBS and cell-associated fluorescence was assessed by the fluorescence plate reader (Victor 3, Perkin Elmer). In a subset of experiments, after 2 h, cells were stained with rhodamine-conjugated phalloidin and green fluorescent A375 cells were identified on multiple z-series collected at 0.20 μm intervals using a confocal microscope (Carl Zeiss).

### Trans-endothelial migration

Trans-endothelial migration assays were performed using the xCELLigence RTCA technology as described described [44]. Briefly, HUVECs (2 × 10^4^ cells/well) suspended in growth medium, were plated on E-16-well plates and allowed to grow for ~25 h until they form a confluent monolayer, prior to seeding melanoma cells (2 × 10^4^ cells/well) in growth medium plus/minus 10 nM RI-3. When HUVECs are challenged with crossing cells, there is a drop in electrical resistance which is monitored in real-time for 5 h as the cell index changes due to crossing of the endothelial monolayer. The experiment was performed twice in quadruplicate.

### 3D organotypic collagen I/fibroblast invasion assay

Organotypic culture system was carried out as described by Timpson and coworkers [48]. Briefly, 1 × 10^5^ NHDF normal, dermal fibroblasts were starved in serum-free medium for 18 h, suspended in 250 μl FBS and embedded in 250 μl alpha Minimum Essential Medium 10× (αMEM 10×) containing 2 mg/mL Type I Collagen (#124–25; Cell Application INC.). Collagen/fibroblast mixture (2.5 ml/well) was plated in 35 mm plastic dishes and allowed to polymerize for 1 h at 37 °C, before the addition of 2 mL growth medium. Collagen/fibroblast matrix was allowed to contract until it fitted in a 24-well dish (~8 days), changing media every other day. Then, 1 × 10^5^ melanoma cells were seeded on top of the matrix and allowed to grow for 72 h, before transferring the matrix to a grid (Screens for CD-1™ size 40 mesh S0770 Sigma) in order to create an air/liquid interface and a chemotactic gradient that promotes cell invasion. Melanoma cells were allowed to invade replacing growth medium, with/without 10 nM RI3, every 2 days. After 14 days, matrixes were cut in half, fixed with 10% formalin and processed for paraffin embedding. Microtome sections about 5 μm thick, were stained with hematoxylin and eosin solutions and analyzed by using a microscope connected to a video camera (Carl Zeiss).

### Statistical analysis

The results are expressed as the means ± standard deviations of the number of the indicated determinations. Data were analyzed by one-way ANOVA and post hoc Dunnett t-test for multiple comparisons. *P* < 0.05 was accepted as significant.

## Results

### Requirement of the uPAR_84–95_ sequence for migration, invasion and trans-endothelial migration of melanoma cells

**Fig. 1:**
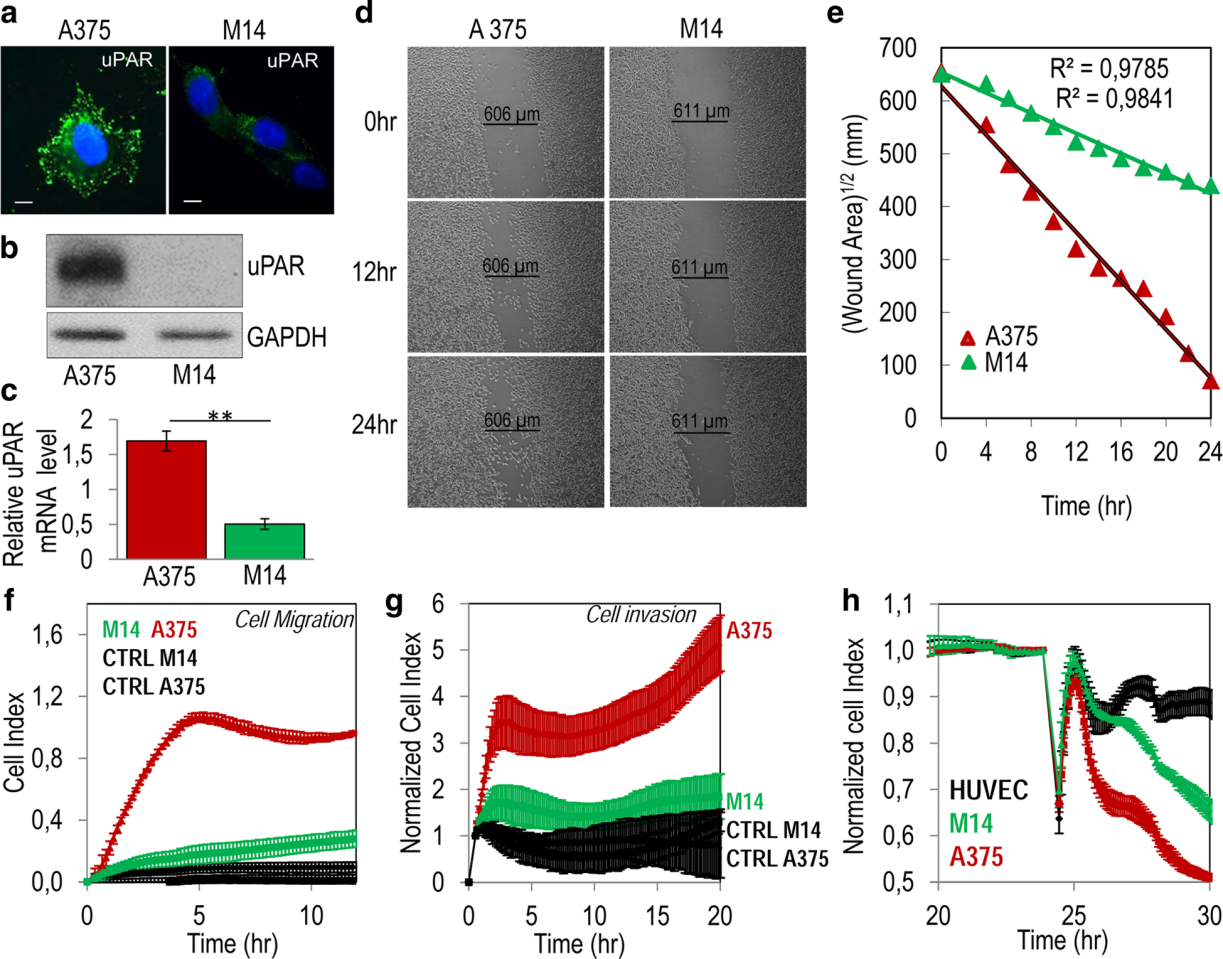
Comparison of migration, matrigel invasion and trans-endothelial migration ability of uPAR expressing A375 and uPAR lacking M14 melanoma cells. **a** Representative images of human A375 and M14 melanoma cells immune-stained with R4 anti-uPAR monoclonal antibody and visualized by a fluorescence inverted microscope. Nuclei were stained blue with DAPI. Scale bar: 5 μm. Original magnification: 1000 x. **b** Whole cell lysates (40 μg/sample) from A375 and M14 cells were resolved on a 10% SDS-PAGE followed by Western blotting with 1 μg/mL R4 anti-uPAR monoclonal antibody or 0.2 μg/mL anti-GAPDH polyclonal antibody as loading control. **c** Quantitative Real-Time PCR of uPAR in A375 and M14 melanoma cell lines. Results are the mean ± SD of three different experiments. **: *p* < 0.001. **d-e** Wound healing of A375 and M14 melanoma cells kept in growth medium at 37 °C, under a 5% CO_2_ atmosphere. One field which includes the scratched path from each dish was selected and scanned sequentially every 30 min for 24 h. Images were recorded at the indicated times by a video-camera connected with a motorized inverted microscope. (Original magnification: 50×). **e** Square root of the wound area measured at the indicated times. **f-g** A375 and M14 cell migration (**f**) or matrigel invasion (**g**) toward serum-free medium (CTRL), or medium containing 10% FBS as source of chemoattractants, monitored for the indicated times by the xCELLigence system. Data represent mean ± SD from a quadruplicate experiment. **h** Trans-endothelial migration of A375 and M14 melanoma cells. HUVECs (1 × 10^4^ cells/well) suspended in growth medium, were allowed to grow for 24 h until they formed a confluent monolayer, prior to seeding melanoma cells (1 × 10^4^ cells/well). The breaking of monolayer integrity was monitored in real-time as changes in Cell Index for additional 5 h. Data represent mean ± SD from a quadruplicate experiment


Our first aim was to investigate the contribution of uPAR to the capability of melanoma cells to respond to chemotactic gradients, invade basement membrane and cross endothelial barriers. As a first approach, we employed the human A375 and M14 melanoma cell lines which express high and low levels of uPAR, respectively. As shown in Fig. 1a R4 anti-uPAR monoclonal antibody appears to react with the overall A375 cell surface, mostly with membrane protrusions, whereas only a punctate, scattered immune-staining was observed on M14 cell surface. Western Blotting and quantitative Real-Time PCR analysis confirmed the expression of uPAR protein and mRNA levels, respectively, in A375 cell lysates but not in M14 cells (Fig. 1b-c and Additional file 1: Figure S1 for full blots). A375 and M14 cell spreading was compared in a wound healing assay monitored for 24 h by time-lapse video microscopy. In the presence of growth medium, A375 cell wounds disappeared after about 24 h, whereas wound repair of M14 did not occur in this time range. Interestingly, computational analysis of wounded area estimated during wound closure, revealed that A375 and M14 cell speed were 0.40 and 0.23 μm/min, respectively (Fig. 1d-e and movies 1 and 2 in the Additional files 2 and 3). The different speed of wound closure by of A375 and M14 cells was not due to a different proliferation rate since doubling index of the two cell lines were comparable (16,7 and 17,3 h, respectively, as shown in the Additional file 4: Figure S2). When cell migration or invasion toward serum, employed as a source of chemoattractants, were monitored in real time for 12 h or 20 h, respectively, by using the xCELLigence technology, we found that A375 cells exhibit an appreciable capability to migrate or cross matrigel, whereas M14 cells did not (Fig. 1f-g). To compare the ability of A375 and M14 melanoma cell lines to cross an endothelial monolayer, endothelial cells were allowed to grow in plates for about 25 h until they formed a monolayer, prior to seeding melanoma cells in the presence of complete medium. At this time, reduction of impedance values, due to invading cells that interrupt monolayers was monitored for further 5 h. Both A375 and M14 cells were able to disrupt the endothelial monolayer although to a different extent. According to migration and matrigel invasion data, A375 cells interrupted endothelial monolayers much more efficiently than M14 cells (Fig. 1h).

Additional file 2: Movie S1.Wound healing assay of A375 melanoma cells. Confluent A375 cells in a 24 multi-well plate were kept in growth medium at 37 °C in a 5% CO_2_ of a Zeiss inverted microscope equipped with a motorized stage. One field which includes the scratched path from each dish was selected and scanned sequentially every 30 min for 24 h. (MOV 3870 kb)
Additional file 3: Movie S2.Wound healing assay of M14 melanoma cells. Confluent M14 cells in a 24 multi-well plate were kept in growth medium at 37 °C in a 5% CO_2_ of a Zeiss inverted microscope equipped with a motorized stage. One field which includes the scratched path from each dish was selected and scanned sequentially every 30 min for 24 h. (MOV 3850 kb)


**Fig. 2:**
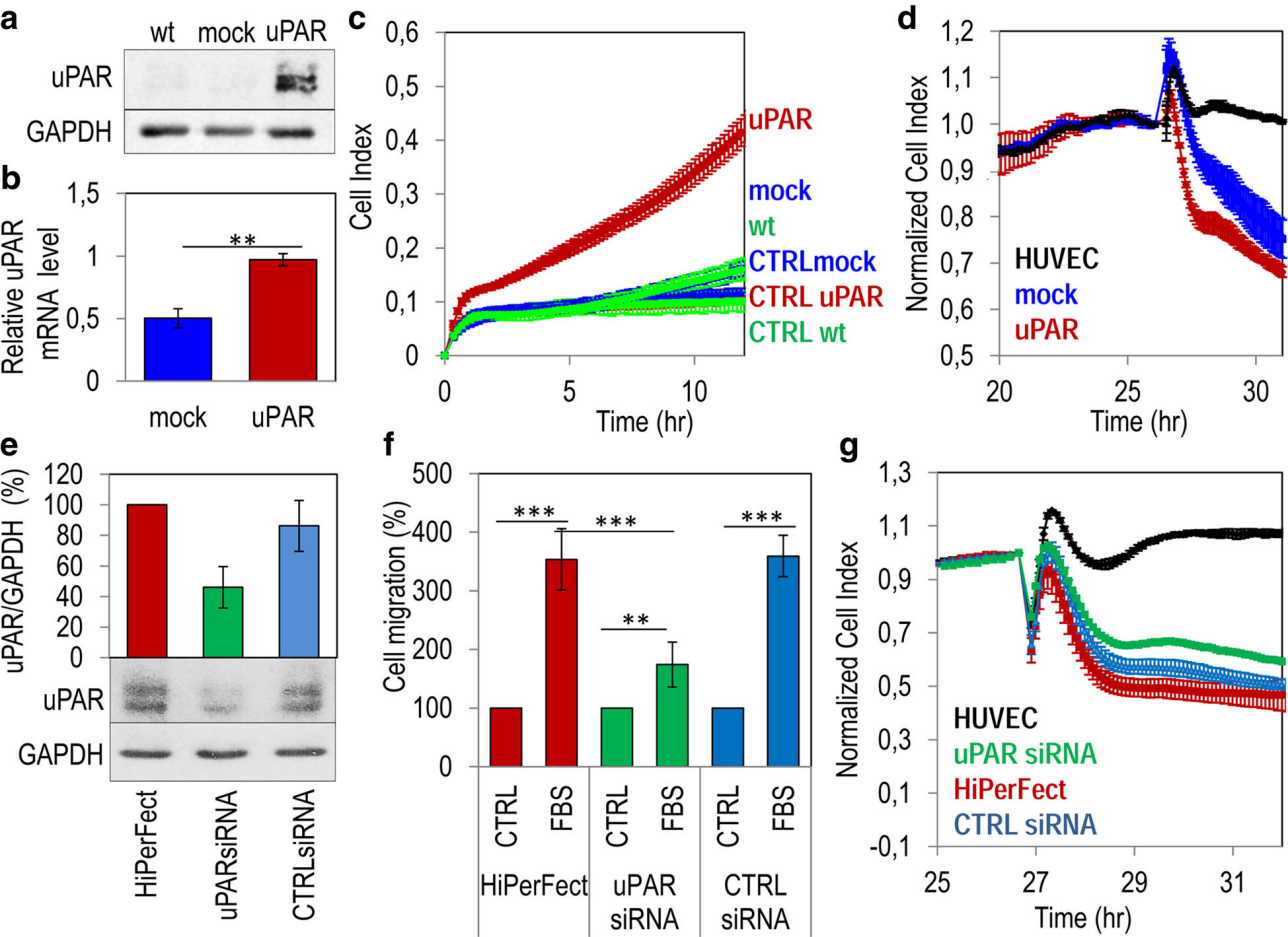
Relevance of uPAR to migration and trans-endothelial migration of melanoma cells. M14 cells were stably transfected with pcDNA3 empty vector (mock) or pcDNA3 loaded with full length uPAR (uPAR). **a** Whole cell lysates (40 μg/sample) from wild type (wt) or transfected M14 cells were resolved on a 10% SDS-PAGE followed by Western blotting with R4 anti-uPAR monoclonal antibody or anti-GAPDH polyclonal antibody as loading control. **b** Quantitative Real-Time PCR of uPAR in mock and in uPAR transfected M14 cells. Results are the mean ± SD of three different experiments. **: *p* < 0.001. **c** Cell migration of wild type (wt), mock and uPAR expressing M14 melanoma cells toward serum-free medium (CTRL), or medium containing 10% FBS monitored for 12 h by the xCELLigence system. Data represent mean ± SD from a quadruplicate experiment. **d** Trans-endothelial migration of wild type, mock and uPAR expressing M14 melanoma cells. Data represent mean ± SD from a quadruplicate experiment. **e** Whole cell lysates (40 μg/sample) from A375 cells transfected with siRNA targeting uPAR (uPARsiRNA), CTRLsiRNA or HyPerFect alone for 96 h were resolved on a 10% SDS-PAGE followed by Western blotting with R4 anti-uPAR monoclonal antibody or anti-GAPDH polyclonal antibody as loading control. The enclosed bar graph shows the average quantification of the uPAR/GAPDH content from 3 independent experiments. **f** Cell migration of A375 cells transfected with uPARsiRNA, CTRLsiRNA or HyPerFect for 96 h. Cells were allowed to migrate for 4 h at 37 °C in 5% CO_2_ in Boyden chambers toward serum-free medium (CTRL) or medium containing 10% FBS (FBS). The extent of cell migration was expressed as a percentage of the basal cell migration assessed in the absence of chemoattractant, considered as 100% (CTRL). Data are expressed as the mean ± SD of three independent experiments, performed in triplicate. Statistical significance with ** p < 0.001, ***p < 0.0001. **g** Trans-endothelial migration of A375 cells transfected with uPARsiRNA, CTRLsiRNA or HyPerFect alone. Data represent mean ± SD from a quadruplicate experiment

It is interesting to note that A375 cell line, exhibiting the most aggressive behavior, is indeed expressing the uPAR at high levels. To ascertain the relevance of uPAR to melanoma cell invasion, we attempted a modulation of uPAR expression and the resulting migratory and invasive phenotype were investigated. First, we overexpressed this receptor in the uPAR-negative M14 cell line. M14 cells were stably transfected with pcDNA3 empty vector (mock) or pcDNA3 carrying cDNA encoding full length uPAR (uPAR). Five G418 resistant clones were analysed by Western blot using the R4 anti-uPAR monoclonal antibody. For functional experiments, we selected the clone #2 (M14/uPAR) which expresses an appreciable amount of uPAR as shown by Western and quantitative Real-Time PCR analysis, as compared to M14/mock cells (Fig. 2a-b, and Additional file 1: Figure S1 for full blots). It has to be taken into account that doubling times of M14 wild type, M14/mock and M14/uPAR, calculated during their exponential growth were quite similar (23.9 h, 23 h and 25,5 h, respectively as shown in the Additional file 4: Figure S2). Using the xCELLigence technology, we found that parental as well as mock-transfected M14 cells exhibit a scarce ability to migrate toward serum, with cell index very similar to the basal levels, recorded in the absence of serum (CTRLwt and CTRLmock). Vice-versa, a dramatic increase of migration was achieved by M14 cells overexpressing uPAR (Fig. 2c). Accordingly, M14/uPAR cells exhibited an increased ability to reduce endothelial monolayer integrity as compared to M14 mock cells (Fig. 2d). Conversely, A375 cells were silenced with uPAR-targeting siRNA. A375cells silenced for uPAR (uPARsiRNA) revealed an about 50% reduction in the uPAR content as shown by Western blot analysis (Fig. 2e and Additional file 1: Figure S1 for full blots). When tested for migration toward serum, A375-uPARsiRNA exhibited an about 50% reduction in their capability to move toward serum, as compared to A375 cells carrying CTRLsiRNA or mock transfected (Fig. 2f). As shown in the Fig. 2g, A375 cells silenced for uPAR disrupted an endothelial monolayer less efficiently as compared to control cells (a 35% reduction was achieved by uPAR silenced A375 cells as compared to cells transfected with the CTRLsiRNA or HiPerFect).

**Fig. 3:**
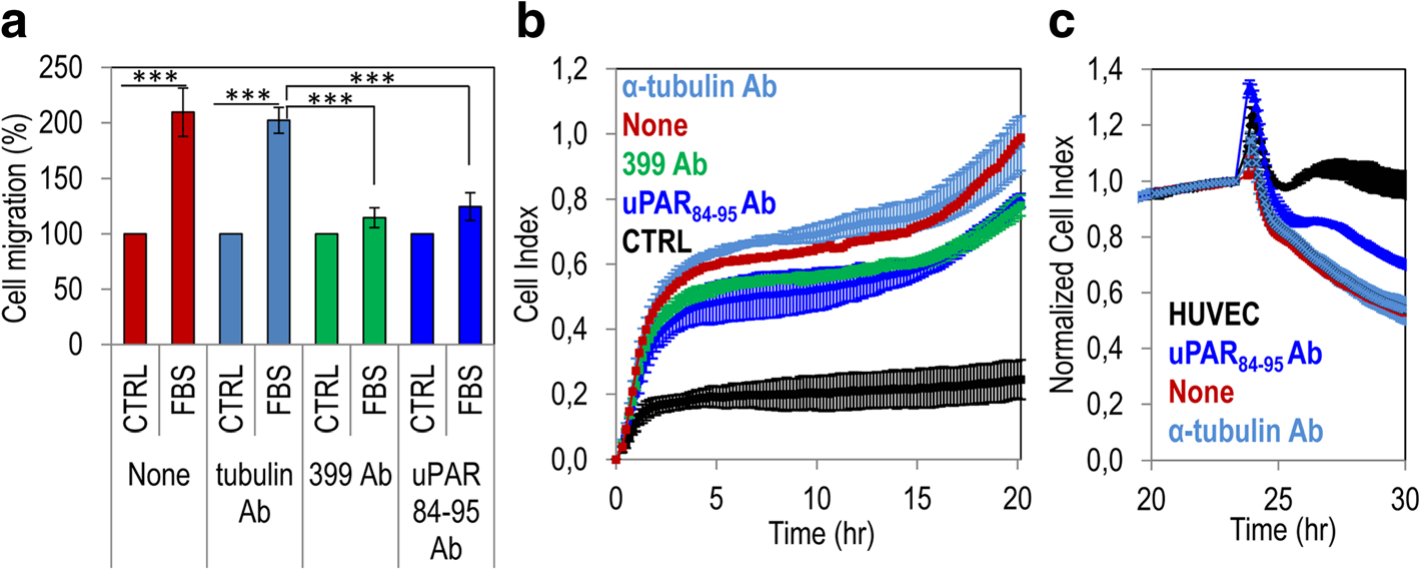
Relevance of the uPAR_84–95_ sequence to migration, matrigel invasion and trans-endothelial migration of melanoma cells. **a** Cell migration of A375 cells in Boyden chambers for 4 h at 37 °C toward serum-free medium (CTRL) or medium containing 10% FBS (FBS) in the presence of diluents (None), or 2 μg/mL the indicated antibodies. For quantitative analysis of cell migration, values were reported as percentage of the basal value assessed in the absence of chemoattractant taken as 100% (CTRL). Data are the means ± SD of three independent experiments, performed in triplicate. Statistical significance with ***p < 0.0001. **b** Matrigel invasion of A375 cells monitored by the xCELLigence system. Cells were seeded on polymerized matrigel and allowed to invade matrigel for 20 h. Lower chambers were filled with serum-free medium (CTRL) or growth medium plus diluents (None) or 2 μg/mL the indicated antibodies. Invasion was monitored in real-time as changes in Cell Index. Data represent mean ± SD from a quadruplicate experiment. **c** Trans-endothelial migration of A375 cells seeded on an endothelial monolayer in the presence of diluents (None), or 2 μg/mL the indicated antibodies. Data represent mean ± SD from a quadruplicate experiment

Furthermore, A375 cell migration was fully prevented by 399 anti-uPAR as well as by anti-uPAR_84–95_ polyclonal antibodies but not by the anti-α-tubulin polyclonal antibody (Fig. 3a). Accordingly, both 399 anti uPAR and anti-uPAR_84–95_ polyclonal antibodies reduced in a comparable manner the extent of matrigel invasion by A375 cells, whereas anti-α-tubulin was ineffective (Fig. 3b). Also, the anti-uPAR_84–95_ Ab reduced the capability of A375 cells to disrupt endothelial monolayers by about 50% (Fig. 3c). Taken together, these finding highlight the potent pro-migratory and pro-invasive abilities of uPAR_84–95_ sequence in melanoma cells.

### Requirement of the uPAR_84–95_-dependent FPR1 activation for migration, invasion and trans-endothelial migration of melanoma cells

**Fig. 4:**
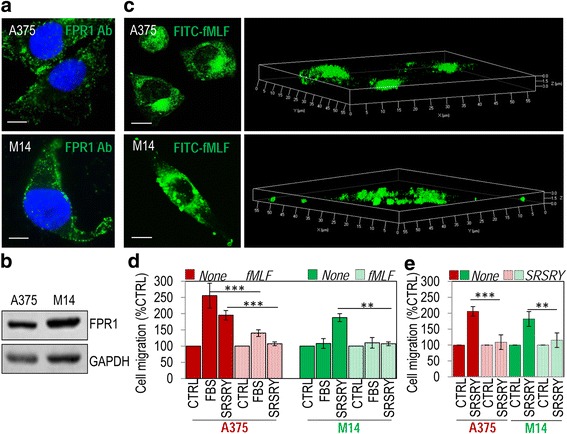
Relevance of the FPR1 to migration of melanoma cells. **a** Representative images of human melanoma A375 and M14 cells immune-stained with anti-FPR1 polyclonal antibody and visualized by a fluorescence inverted microscope. Nuclei were stained blue with DAPI. Scale bar: 5 μm. Original magnification: 1000 x. **b** Whole cell lysates (40 μg/sample) from A375 and M14 cells were resolved on a 10% SDS-PAGE followed by Western blotting with 1 μg/mL anti-FPR1 polyclonal antibody or 0.2 μg/mL anti-GAPDH polyclonal antibody as loading controls. **c** A375 and M14 melanoma cells exposed to 10 nM FITC-fMLF for 30 min at 37 °C and then visualized using a Zeiss 510 Meta LSM microscope in 2D (left) or 3D (right) projections. Scale bar: 5 μm. Original magnification: 630×. **d-e** A375 (red) and M14 (green) melanoma cells were exposed to diluents (None), or desensitized (dotted boxes) with 100 nM fMLF (**d**) or 100 nM SRSRY (**e**) for 1 h at 37 °C and then allowed to migrate in Boyden chambers for 4 h at 37 °C toward serum-free medium (CTRL), 10% FBS or 10 nM SRSRY. The basal value assessed in the absence of chemoattractant (CTRL) was taken as 100% and all values were reported relative to that. Data are the means ± SD of three independent experiments, performed in triplicate. Statistical significance with **p < 0.001 and *** *p* < 0.0001

We and others have previously documented that: ì) FPR1 desensitization with an excess of fMLF makes FPR1 unavailable on cell surface, thus interfering with receptor activation and subsequent cell migration [22, 31, 32]; ìì) uPAR binds to FPR1 through its Ser^88^-Arg-Ser-Arg-Tyr^92^ sequence, thus promoting FPR1 internalization which is essential for cell migration [31]; ììì) the minimal 88–92 sequence of uPAR triggers directional cell migration also in the form of a synthetic peptide [22, 31]; iv) uPAR lacking and FPR1 expressing HEK-293 cells move toward the chemotactic sequence of uPAR as well as to SRSRY peptide, their movement being abrogated by FPR1 desensitization also with an excess of SRSRY [22, 38]. First we assessed whether A375 and M14 cells express FPR1 and whether agonist-dependent FPR1 internalization does occur following exposure to 10 nM N-formyl-Nle-Leu-Phe-Nle-Tyr-Lys-fluorescein (FITC-fMLF) as described [39, 40]. Both A375 and M14 cells express considerable levels of FPR1 as shown by immunofluorescence and Western blot analysis (Fig. 4a-b and Additional file 1: Figure S1 for full blots)*.* FITC-fMLF-dependent FPR1 internalization does occur in both A375 and M14 cells as cell exposure to the fluorescent agonist for 30 min at 37 °C caused the appearance of intra-cytoplasmic green fluorescent spots (Fig. 4c). 3D reconstruction of z-stack analysis confirmed that the internalization of FPR1 does occur in both melanoma cell lines (Fig. 4c, right). As expected, A375 cell motility toward serum as well as toward SRSRY dramatically decreased upon FPR1 desensitization with an excess of fMLF (Fig. 4d) or SRSRY (Fig. 4e). In contrast, although expressing FPR1, M14 cells, are unable to migrate toward serum, but retain the ability to migrate toward SRSRY, the last being abrogated by FPR1 desensitization with an excess of fMLP (Fig. 4d) or SRSRY (Fig. 4e). These findings indicate that FPR1 is necessary but not sufficient to elicit cell motility and that the potency of uPAR_84–95_ to promote melanoma cell ability to migrate, is mainly mediated by FPR1. Thus, the uPAR_84–95_/FPR1 complex may be considered an attractive therapeutic target for melanoma cells.

### Targeting the uPAR_84–95_/FPR1 cross-talk for preventing migration and matrigel invasion of melanoma cells

**Fig. 5:**
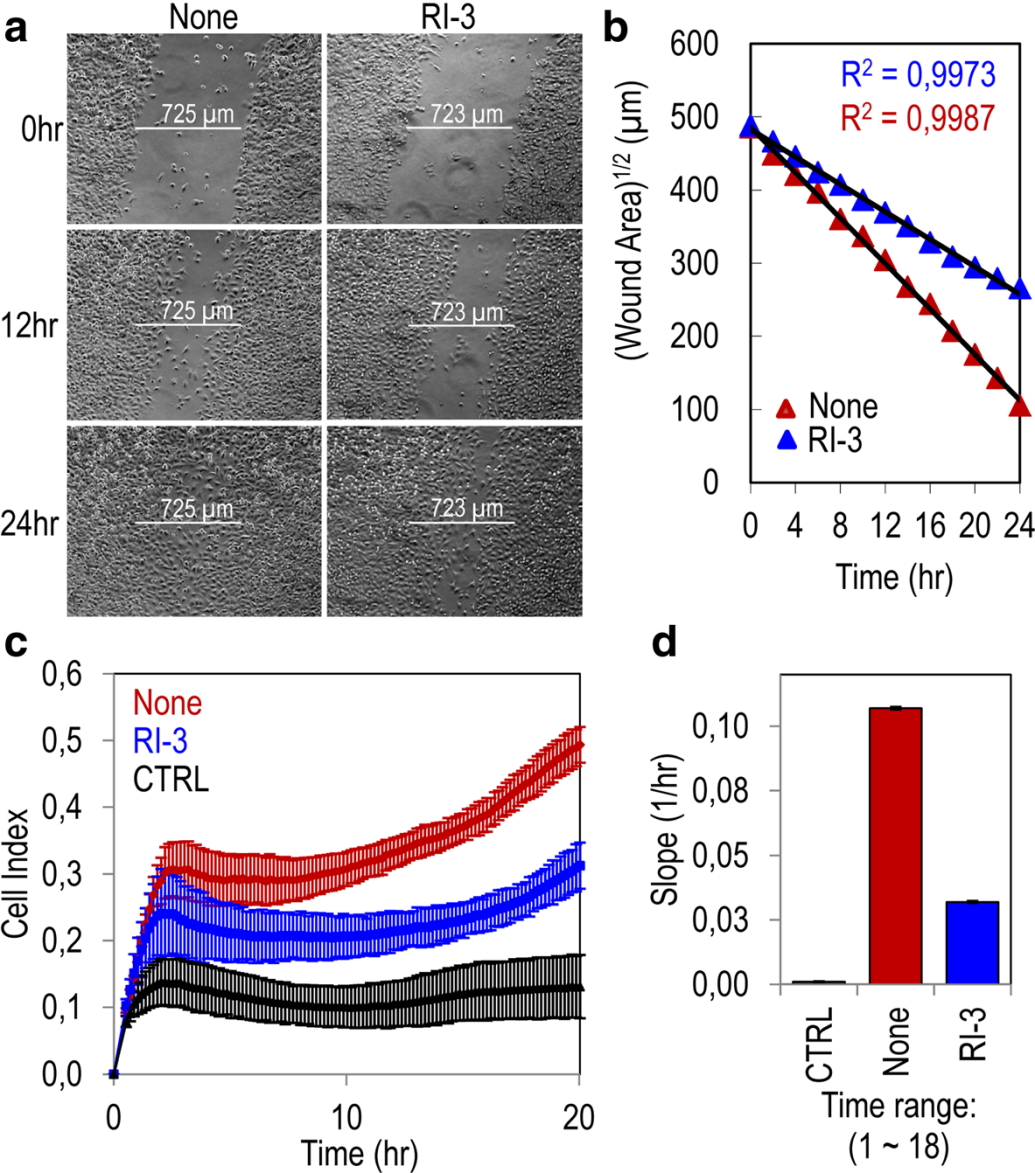
RI-3 inhibits migration and matrigel invasion of A375 melanoma cells. **a** Wound healing of A375 melanoma in the absence (None) or presence of 10 nM RI-3. Cells were kept in growth medium at 37 °C in a 5% CO_2_ of a Zeiss inverted microscope equipped with a motorized stage. One field which includes the scratched path from each dish was selected and scanned sequentially every 30 min for 24 h. Images were recorded at the indicated times by a video-camera connected with a motorized inverted microscope. Original magnification: 50×. **b** Square root of the wound area measured at the indicated times. **c** Matrigel invasion of A375 cells monitored by the xCELLigence system for 20 h in the presence or the absence of 10 nM RI-3. Data represent mean ± SD from a quadruplicate experiment. **d** Slopes represent the change rate of Cell Indexes generated in a 1–18 h time frame


Previous work from this laboratory showed that substitution of Ser90 in full length, membrane-associated uPAR, affects the complex uPAR/FPR1 cross-talk [38]. In the past years we developed a family of peptides containing the Arg-Glu-Arg or the Arg-Aib-Arg central core. They share the same binding site with the chemotactic sequence, prevent uPAR/FPR1 interaction and inhibit cell migration, invasion and angiogenesis [39–42]. More recently, starting from the lead peptide N-terminal acetylated and C-terminal amidated Ac-Arg-Glu-Aib-Tyr-NH_2_ peptide, we applied the Retro-Inverso (RI) approach to develop a new family of enzyme-resistant analogues. Among these, we selected the peptide Ac-(D)-Tyr-(D)-Arg-Aib-(D)-Arg-NH_2_ (RI-3) which is a nanomolar inhibitor of uPAR_84–95_-dependent, FPR1-mediated signaling [44]. Thus, we investigated the possibility that RI-3 would inhibit migration, extracellular matrix invasion and trans-endothelial migration of A375 cells expressing uPAR and FPR-1. In a wound healing assay monitored for 24 h by time-lapse video microscopy, RI-3 caused a 1.7-fold reduction in the cell speed of A375 cells exposed to growth medium plus 10 nM RI-3 as compared to those exposed to growth medium plus diluents (0.154 vs.0.264 μm/min, respectively, (Fig. 5a-b and movies 3 and 4 available in the Additional files 5 and 6). Furthermore, matrigel invasion by A375 was dramatically reduced in the presence of RI-3 (Fig. 5c). These differences appeared more evident when slopes, which represent the rate of change of the Cell Index, were generated in the 1–18 h range. A 60% reduction in the ability of A375 cells to cross matrigel was achieved by 10 nM RI-3 (Fig. 5d).

Additional file 5: Movie S3.Wound healing assay of A375 melanoma cells exposed to diluents. Confluent A375 cells in a 24 multi-well plate were kept in growth medium at 37 °C in a5% CO_2_ of a Zeiss inverted microscope equipped with a motorized stage. One field which includes the scratched path from each dish was selected and scanned sequentially every 30 min for 24 h. (MOV 4350 kb)
Additional file 6: Movie S4.Wound healing assay of A375 melanoma cells exposed to RI-3. Confluent A375 cells in a 24 multi-well plate were kept in growth medium in the presence of 10 nM RI-3 at 37 °C in a5% CO_2_ of a Zeiss inverted microscope equipped with a motorized stage. One field which includes the scratched path from each dish was selected and scanned sequentially every 30 min for 24 h. (MOV 3500 kb)


### Targeting the uPAR_84–95_/FPR1 cross-talk to prevent adhesion to endothelium and trans-endothelial migration of melanoma cells

**Fig. 6:**
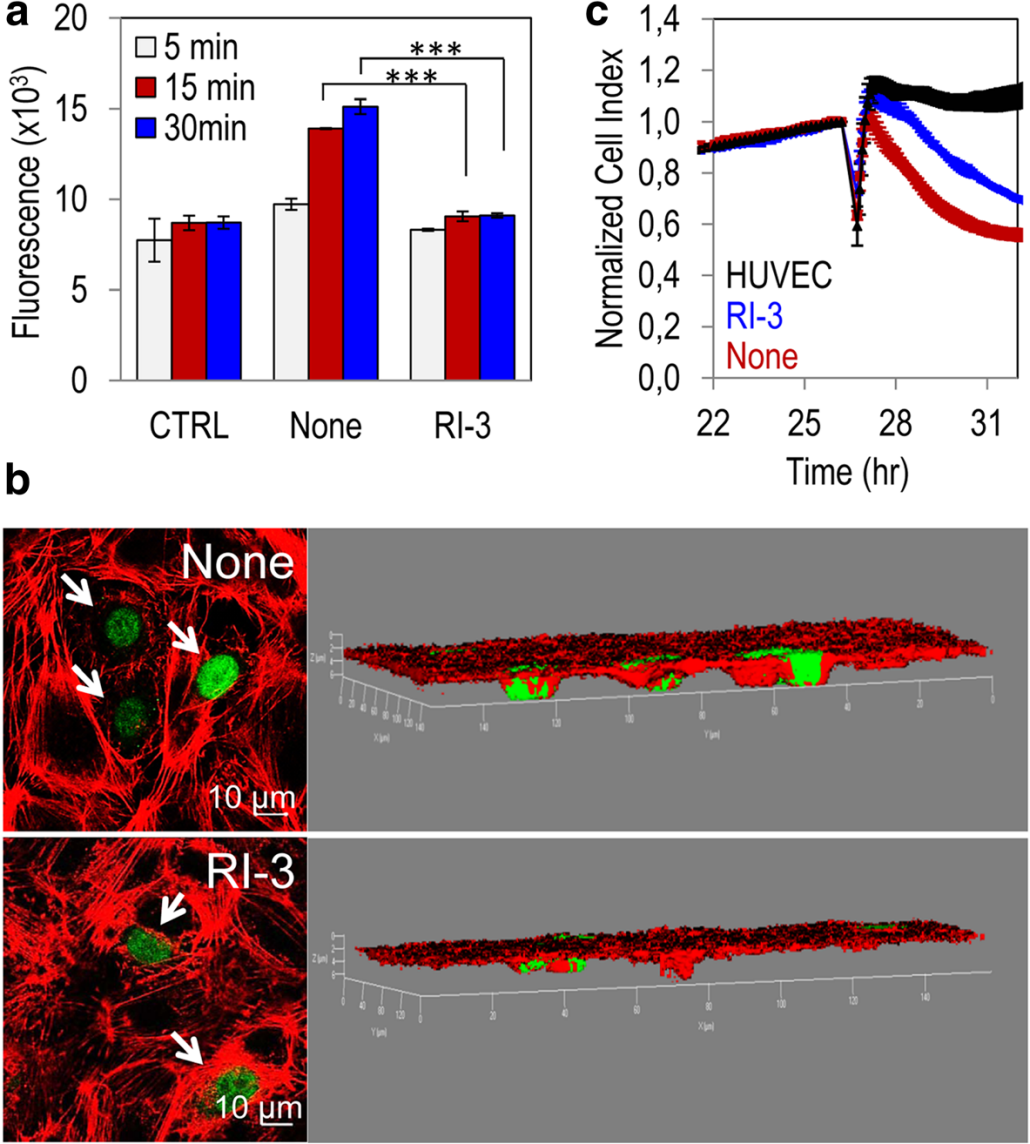
RI-3 prevents in vitro adhesion to endothelium and trans-endothelial migration of melanoma cells. **a** HUVEC were seeded onto matrigel and allowed to attach and grow for 24 h (CTRL) prior to seeding GFP-A375 cells suspended in complete endothelial medium plus diluents (None), or 10 nM RI-3 at 37 °C, 5% CO_2_. At the indicated times, cell associated fluorescence was assessed by a fluorescence plate reader. Data represent means ± SD of three independent experiments performed in duplicate. Statistical significance with ***p < .0.0001. **b** After 2 h, cells were stained with rhodamine-phalloidin and GFP-A375 cells (arrows) visualized on multiple z-series collected at 0.20 μm intervals by laser confocal microscopy. On the left representative images recorded in 3D projection are shown. Original magnifications: 400×. **c** Trans-endothelial migration of A375 cells. HUVECs (1 × 10^4^ cells/well) suspended in growth medium, were grown until they formed a confluent monolayer, prior to seeding A375 cells (1 × 10^4^ cells/well) in growth medium plus diluents (None) or 10 nM RI-3. Data represent mean ± SD from a quadruplicate experiment

The attachment of tumor cells to the endothelium and their entry into bloodstream are early events occurring during the metastatic process. To ascertain if RI-3 influences tumor cell adhesion to the endothelium, GFP-tagged A375 cells were seeded onto an endothelial monolayer in the presence/absence of 10 nM RI-3. At the indicated times, non-adherent cells were removed and the cell associated fluorescence was measured using a fluorescence plate reader. For each time point, the fluorescence values associated to endothelial cells alone (CTRL) were assessed. Already after 5–10 min of incubation, we found an appreciable adhesion of GFP-A375 cells to endothelium, that increased with time. After 5, 15, and 30 min, 10 nM RI-3 reduced fluorescence by 15%, 35%, and 40%, respectively (Fig. 6a). When the experiment was carried out for 2 h and co-cultures were labeled for F-actin, the analysis of planes confocal to the endothelium, revealed A375 cells interacting with HUVECs, that decreased in the presence of 10 nM RI-3 (Fig. 6b, arrows). Z-stack analysis of the images recorded with 0.20 μm intervals through the entire thickness of the endothelial monolayer and visualized in 3D projection, confirmed that the majority of melanoma cells are confocal to or below the endothelial monolayer in the absence of any treatment. When RI-3 was added to the co-cultures at 10 nM concentration, the majority of melanoma cells rested on the plane of endothelial cells (Fig. 6b). These data indicate that RI-3 prevents the attachment of tumor cells to endothelium, and suggest that RI-3 may also reduce trans-endothelial migration of tumor cells. Therefore, the ability of A375 cells to cross an endothelial monolayer was analyzed in the presence or the absence of 10 nM RI-3, using the xCELLigence technology. As expected, an appreciable reduction of endothelial monolayer integrity was achieved with A375 cells. We found that 10 nM RI-3 effectively reduced the capability of melanoma cells to disrupt endothelial monolayers (Fig. 6c). These data indicate that RI-3 prevents the adhesion of tumor cells to endothelium, and reduces trans-endothelial tumor cell migration.

### RI-3 peptide prevents A375M6 melanoma cell ability to invade fibroblast-embedded collagen matrices

**Fig. 7:**
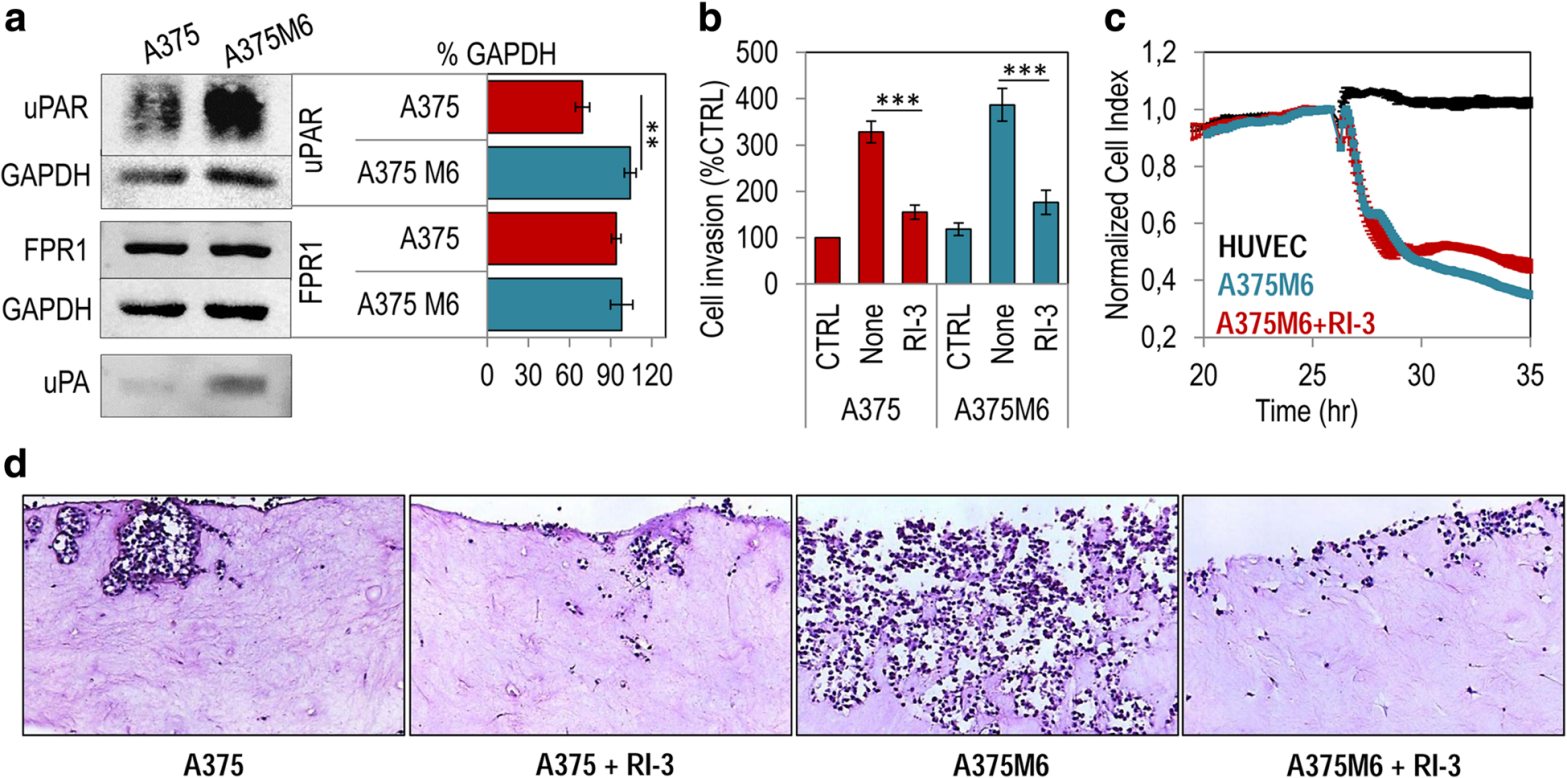
RI-3 prevents melanoma cell invasion through collagen I matrices contracted by dermal fibroblasts. **a** Whole cell lysates (40 μg/sample) of A375 or A375M6 melanoma cells were resolved on a 10% SDS-PAGE followed by Western blotting with 1 μg/mL R4 anti-uPAR monoclonal antibody, 1 μg/mL anti-FPR1 polyclonal antibody or 0.2 μg/mL anti-GAPDH polyclonal antibody, the last as loading controls. *Lower panel:* 50 μl of concentrated conditioned medium from A375 and A375M6 cells were resolved on a 10% SDS-PAGE under unreducing conditions followed by Western blotting with 1 μg/mL 389 anti-uPA polyclonal antibody. Bar graphs showing the average quantification of the uPAR/GAPDH and FPR1/GAPDH content from 3 independent experiments. Statistical significance with **p < 0.001. **b** A375 and A375M6 melanoma cells were allowed to invade matrigel for 18 h in Boyden chambers toward serum-free medium (CTRL) or medium containing 10% FBS (FBS), in the absence (None) or the presence of 10 nM RI-3. The extent of cell invasion was expressed as a percentage of the A375 basal cell invasion assessed in the absence of chemoattractant, considered as 100% (CTRL). Data are expressed as the mean ± SD of three independent experiments, performed in triplicate. Statistical significance with ***p < 0.0001. **c** Trans-endothelial migration of A375 and A375M6 cells plus/minus 10 nM RI-3. Data represent mean ± SD from a quadruplicate experiment. **d** Photographs showing A375 and A375M6 cell invasion of collagen I matrices contracted by dermal fibroblasts in the absence or presence of 10 nM RI-3. Original magnification. 100×

To further analyze the efficacy of RI-3 to counteract invasion of ECM and endothelium by melanoma cells, we took advantage by using A375 derived metastatic M6 cells that are documented to express higher levels of uPAR on cell surface and exhibit a more robust invasive ability then A375 cells [49]. Western blot analysis of cell lysates confirmed that A375M6 cells express higher levels of uPAR than A375 cells (about a 1.5-fold increase in the uPAR content) and comparable levels of FPR1 (Fig. 7a and Additional file 1: Figure S1 for full blots). In keeping with their higher invasive capability, a larger amount of uPA was found in conditioned medium from A375M6 as compared to A375 cells (Fig. 7a and Additional file 1: Fig. S1 for full blots). Not surprisingly, 10% FBS elicited a considerable cell invasion of A375 and A375M6 cells, reaching 328% and 387% of the basal cell invasion, respectively. In a Boyden chamber assay, the addition of 10 nM RI-3 to the lower compartment, reduced matrigel invasion of A375 and A375M6 cells to a similar extent (53 and 55%, respectively) (Fig. 7b). Furthermore, a dramatic disruption of endothelial monolayer was achieved by A375M6 cells and partially prevented by RI-3 (Fig. 7c). Finally, the effects of RI-3 antagonism were examined in a 3D organotypic in vitro model of invasion that more accurately recapitulates key aspects of the architecture and histology of solid cancers. A375 and A375M6 cells were seeded onto collagen I matrices previously combined with dermal fibroblasts, transferred to an air-liquid interface and allowed to invade the underlying matrix. Growth medium, with/without 10 nM RI-3 was replaced every other day. After 14 days, matrices were fixed in buffered formalin and processed for paraffin sectioning and hematoxylin-eosin staining. Both A375 and A375M6 cells were able to enter into matrices (Fig. 7d). However, the majority of A375M6 deeply invaded the collagen I matrices contracted by dermal fibroblasts (Fig. 7d). These effects are proliferation-independent, because the doubling time of less invasive A375 cells is shorter as compared to that of more invasive A375M6 cells (16,6 h and 23,4 h, respectively) (Additional file 4: Figure S2). In both cases, the presence of 10 nM RI-3 resulted in a significant reduction in the ability of cells to invade matrices (Fig. 7d).

## Discussion

Melanoma is an extremely complex disease, with many mutations in genes governing different signaling pathways. Although the recent targeted- and immune-therapies have significantly prolonged patient survival, effective treatments for metastatic melanoma are lacking to date, and the prognosis for these patients remains very poor. The main focus of this study was to elucidate the role of the uPAR-FPR1 cross-talk in sustaining melanoma cell ability to invade extracellular matrix and cross endothelial barriers, focusing on the function of the uPAR_84–95_ sequence which we and others have previously reported to signal trough FPR1 [21, 22, 31].

In this study, we show for the first time that the uPAR ability to trigger migration, invasion and trans-endothelial migration of melanoma cells involves the internalization of FPR. Regarding the relationship between uPAR and FPR1, we found that FPR1 is necessary but not sufficient to elicit such effect as FPR1-triggered cell motility may occur only in the presence of the uPAR chemotactic sequence for the following reasons: ì) uPAR and FPR1 expressing melanoma cells are able to cross matrigel and interrupt monolayer integrity of endothelial cells, the effects being reduced by antibodies recognizing the uPAR_84–95_ sequence or by the RI-3 peptide, which specifically inhibits uPAR_84–95_-triggered, FPR1-mediated signals; ìì) uPAR lacking but FPR1 expressing M14 cells do migrate toward SRSRY, their motility being reduced to basal levels by cell pre-exposure to an excess of fMLF or SRSRY. Thus, the uPAR_84–95_ region is strongly involved in these steps and requires FPR1 to function. In fact, both uPAR expressing and uPAR lacking melanoma cells respond to FPR1 agonist SRSRY, their motility being abrogated by FPR1 desensitization. Importantly, uPAR is able to trigger FPR1 activation not only in autocrine but also paracrine manner. Indeed, upon plasmin- or uPA-dependent cleavage, soluble forms of uPAR, containing the chemotactic sequence, have been documented to be secreted in the extracellular milieu and promote migration of FPR1 expressing cells [31, 50]. The relevance of this observation is not obvious considering that: i) FPR1 is overexpressed in human primary melanoma and associates with aggressive phenotype [35]; iì) In human glioblastoma and neuroblastoma cells, FPR1 promotes cell growth, invasion and production of angiogenic factors [37]; ììì) In glioblastoma, FPR1 exploits the function of EGFR to promote tumor progression by increasing the phosphorylation on Tyr^992^ in the intracellular tail of EGFR [51]. Since glial cells, and melanocytes have a common pluripotent progenitor [52], the functions of FPR1 might be similar in these cell types.

Like other uPAR_84–95_ sequence-derived peptides previously studied by us, RI-3 adopts the turned structure typical of the previously described linear peptide antagonists of uPAR-FPR1 interaction, is stable in human serum and is a nanomolar competitor of N-formyl-Met-Leu-Phe for binding to FPR1 [44]. However, the selective impairment of uPAR-mediated FPR1 triggered signaling is not expected to affect other functions regulated by FPR1. We have previously shown that peptide inhibitors of the uPAR/FPR1 interaction prevent phosphorylation of p38α and ERK1/2, without affecting intracellular calcium mobilization [39, 41]. RI-3 alone does not elicit any cell response and does not affect cell proliferation in vitro*.* Also, it was apparently well tolerated in vivo when administered to mice with no visible side-effects and no change of body weight vs. vehicle-treated animals [44].

During malignant progression, tumor cells acquire the ability to invade the surrounding tissue and/or spread into distant organs. Invadopodia extend into the ECM and are believed to be important for tumor cell invasion and also intravasation, facilitating intravascular dissemination and metastasis. A number of studies have highlighted molecular targets that control the ability of cancer cells to adapt to the environment by regulating plasticity of cancer cells [53]. Together with the many so far reported pro-tumoral activities of uPAR, our observations make the uPAR/FPR1 system an attractive target for the treatment of melanoma that has not yet been extensively explored in the clinic. Furthermore, uPAR inhibitors have been described to be efficacious also in melanoma cells with acquired resistance to BRAF and MEK inhibitors [13]. Using uPAR-lacking and uPAR-expressing melanoma cells, we found that melanoma cell motility as well as their capability to cross extracellular matrices in the presence of serum is mostly due to the uPAR. Importantly, we found for the first time that uPAR plays an important role in favoring cell adhesion to endothelium and trans-endothelial migration of melanoma cells. Indeed, inhibition of uPAR via RNA interference elicited a dramatic reduction of migration, invasion and trans-endothelial migration of melanoma cells. This is not surprising, as the uPAR, is a molecular mediator of plasticity in cancer cell migration by regulating contractile forces through the functional axis uPAR-integrins-actin [49].

Available data indicate that a successful strategy to combat intravasation and metastatic diffusion of aggressive cancer cells include integrin αvβ3 antagonists [54]. Melanoma cells express high levels of αvβ3 integrin which has been linked to the progression of disease, probably favoring trans-endothelial migration of tumor cells [55, 56]. We have previously documented that the peptide SRSRY promotes cell migration by interacting with FPR1 which, in turn, activates the αvβ3 integrin with an inside-out type of mechanism and that peptide inhibitors of the uPAR/FPR1 interaction disrupt the integrin/FAK signaling pathway [22, 41]. Peptide antagonists of αv integrin subunit are derived from the connecting peptide region of human uPA and are known to prevent tumor cell migration and invasion [57]. It will be interesting to investigate the possibility that combining RI-3 and αvβ3 integrin inhibitors could better prevents metastatic dissemination of melanoma cells.

The experiments reported here and supporting the efficacy of RI peptide have been performed in culture. However, they have been validated in 3-D organotypic assays, where melanoma cell invasion in the presence of stromal fibroblasts was successfully prevented. A number of studies have demonstrated that cellular behavior in 3D cultures rather than 2D culture occur more similarly to those in vivo [58]. 2D cell culture does not adequately take into account the natural 3D environment of cells, particularly in terms of cell-cell interactions and cell-ECM interactions. In addition, the flat substrate in 2D culture imposes the highly unnatural geometric and mechanical constraints on cells; and culturing of cells is limited to single cell types.

Thus, in order to recapitulate key events in invasion, thereby re-establishing morphological and functional features of the corresponding tissue in vivo, 3D organotypic co-cultures are a good option compared to the in vivo assays. In 3D assays, we found that the highly invasive A375M6 cells, exhibit the highest capability to invade collagen I matrices contracted by dermal fibroblasts. Interestingly, A375M6 express higher levels of uPAR and secrete larger amount of uPA as compared to A375 cells. Therefore, we can speculate that A375M6 cells bind uPA focusing proteolytic activity on cell surface.

Melanoma cells display an inherent ability to switch between modes of migration: a mesenchymal-type movement that requires extracellular proteolysis and an amoeboid movement that requires high Rho-kinase signaling and is less dependent on proteases [59]. Although in this study we did not investigate the role of uPA, it is likely that uPA favors mesenchymal-type migration in two ways: ì) upon receptor engagement, uPA converts plasminogen into plasmin, which activates pro-MMPs on cell surface and stimulates uPAR-dependent signaling, activating ERK ½; ìì), plasmin generated by uPA or uPA itself cleaves full uPAR (D1D2D3), leaving the GPI-anchored D2D3 which is reported to support only the mesenchymal-type movement [49]. The uPA-dependent pericellular proteolytic activity may represent a switch between amoeboid and mesenchymal migration styles which are used in different ways to achieve cell motility and often rely on the same intracellular components [60]. Accordingly, Jo and coworkers documented that both Ras-ERK and Rho-Rho kinase pathways cooperate to promote cell migration in uPA-stimulated cells [61]. Since RI-3 fully prevents invasion of both A375 and A375M6 melanoma cells, it is difficult to pinpoint the exact sequence of events and the contribution of each subset of mediators in the inhibitory effects exerted by RI-3. This point will deserve further investigations.

There is increased awareness that cancer therapy should include, in addition to treatment of the primary tumor and established metastases, also the prevention of metastasis formation [62]. These results for melanoma are significant, because the proportion of patients who have metastases at the time of the initial diagnosis is high, and they have poor prognosis. For these patients, combining current therapy with a systemic anti-metastatic agent might considerably improve the outcome.

## Conclusions

Collectively, our findings identify uPAR and FPR1 as novel prognostic markers and therapeutic targets in melanoma and indicate that inhibitors of the uPAR_84–95_/FPR1 cross-talk may be useful for the treatment of metastatic melanoma.

## Additional files

Additional file 1: Figure S1. Uncropped images of immunoblots. Full blots from Fig. 1b, Inset of the Fig. 2a, c, Fig. 4b, and Fig. 7a. (PDF 164 kb)

Additional file 4: Figure S2. Proliferation rate of melanoma cells. Cell proliferation of the indicated melanoma cell lines assessed by monitoring impedance by RTCA xCELLigence system. The reported doubling times were calculated from the cell growth curves, during exponential growth. Data represent mean ± SD from a quadruplicate experiment representative of 3 replicates. (PDF 120 kb)

## Abbreviations

ECM: Extracellular matrix
FBS: Fetal bovine serum
fMLF: N-formyl-Met-Leu-Phe
FPR: Formyl-peptide receptor
GFP: Green fluorescent protein
HUVEC: Human umbilical vein endothelial cell
PBS: Phosphate-buffered saline
RTCA: Real time cell analysis
uPAR: urokinase receptor
