# Supplementary figures and images for: Targeting the cross-talk between Urokinase receptor and Formyl peptide receptor type 1 to prevent invasion and trans-endothelial migration of melanoma cells
Source: J Exp Clin Cancer Res. 2017 Dec 8;36:180. doi: 10.1186/s13046-017-0650-x (PMC5721612; doi:10.1186/s13046-017-0650-x)

Supplementary Figure S1. Uncropped images of immunoblots from Fig.1B,2A 2E,4B and Fig.7A

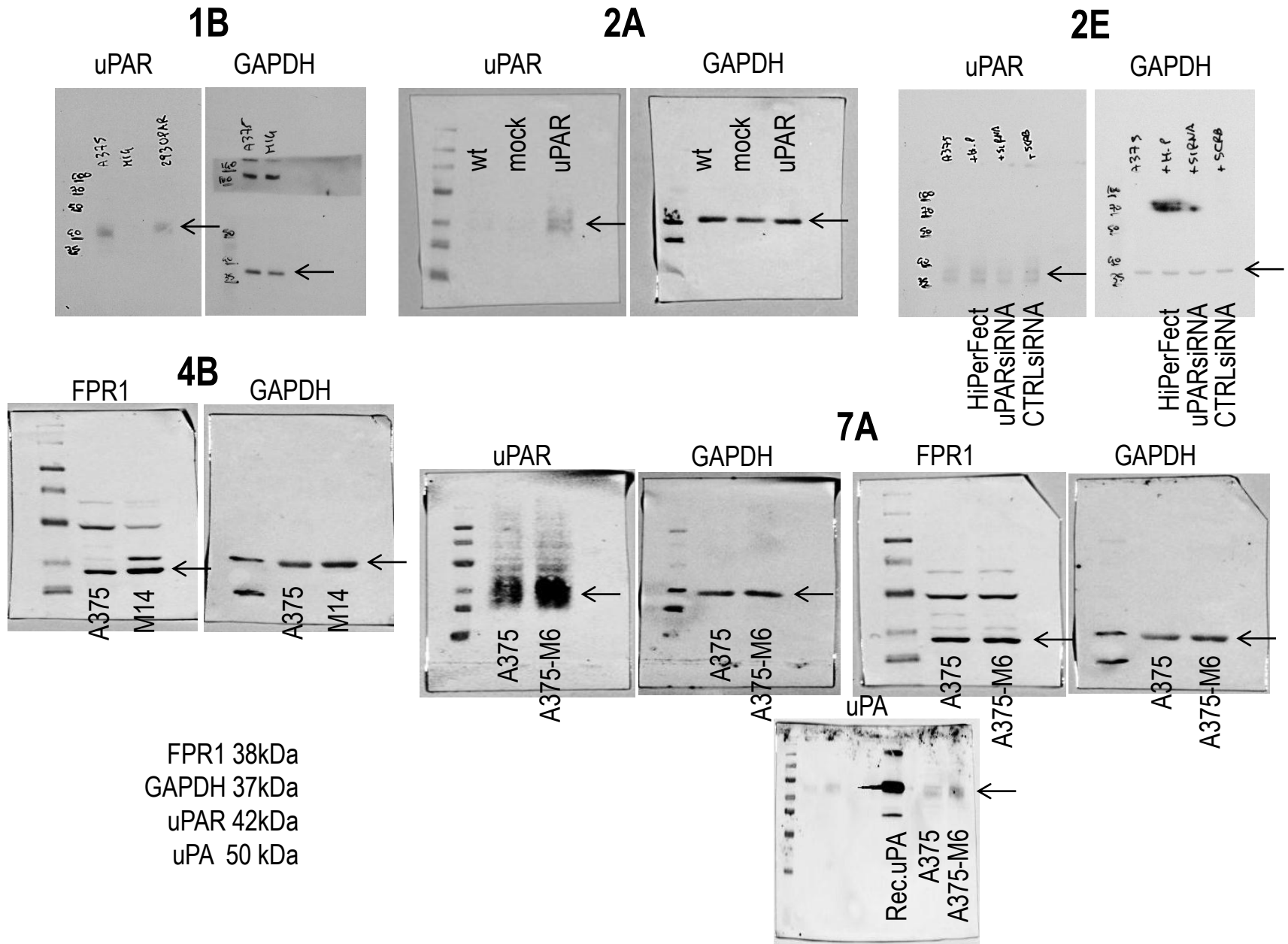

Supplement: Supplementary file 5 — Uncropped images of immunoblots. Full blots from Fig. 1b, Inset of the Fig. 2a, c, Fig. 4b, and Fig. 7a. (PDF 164 kb) [file 13046_2017_650_MOESM1_ESM.pdf]
