# Supplementary material for: Targeting the cross-talk between Urokinase receptor and Formyl peptide receptor type 1 to prevent invasion and trans-endothelial migration of melanoma cells
Source: J Exp Clin Cancer Res. 2017 Dec 8;36:180. doi: 10.1186/s13046-017-0650-x (PMC5721612; doi:10.1186/s13046-017-0650-x)
Supplement: Supplementary file 6 — Proliferation rate of melanoma cells. Cell proliferation of the indicated melanoma cell lines assessed by monitoring impedance by RTCA xCELLigence system. The reported doubling times were calculated from the cell growth curves, during exponential growth. Data represent mean ± SD from a quadruplicate experiment representative of 3 replicates. (PDF 120 kb) [file 13046_2017_650_MOESM4_ESM.pdf]

# Supplementary Figure S2: Proliferation rate of melanoma cells

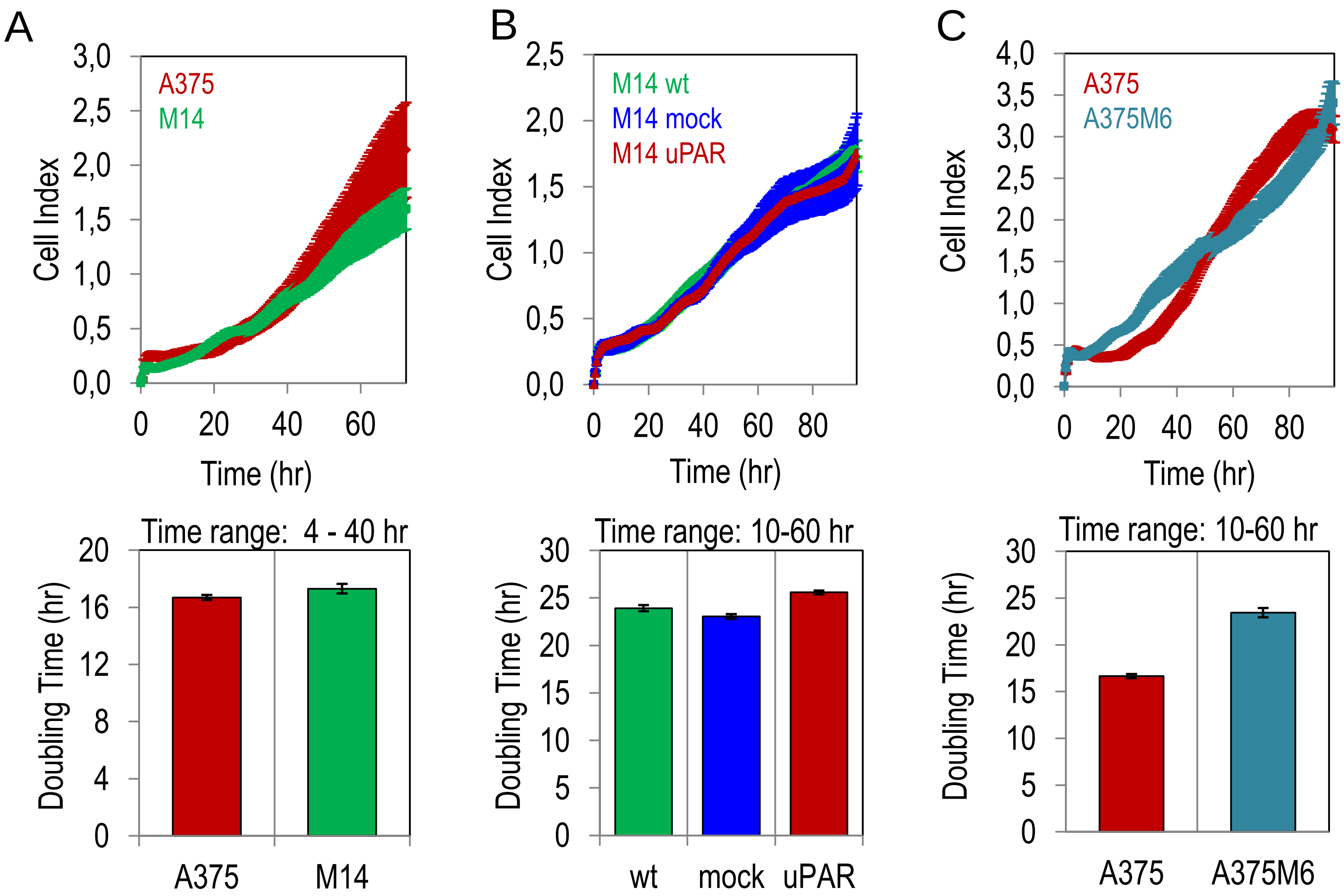


Cell proliferation of the indicated melanoma cell lines assessed by monitoring impedance by RTCA xCELLigence system. The reported doubling times were calculated from the cell growth curves, during exponential growth. Data represent mean ± SD from a quadruplicate experiment representative of 3 replicates.
